# Estrogen receptor targeting with genistein radiolabeled Technetium-99^m^ as radiotracer of breast cancer: Its optimization, characterization, and predicting stability constants by DFT calculation

**DOI:** 10.1016/j.heliyon.2023.e13169

**Published:** 2023-01-21

**Authors:** Danni Ramdhani, Nita Listiani, Maula Eka Sriyani, Eva Maria W, Hiroshi Watabe, Resmi Mustarichie, Sri Agung Fitri Kusuma, Regaputra S. Janitra

**Affiliations:** a Department of Pharmaceutical Analysis and Medicinal Chemistry, Padjadjaran University, Sumedang, West Java, 45363, Indonesia; b Applied Nuclear Science and Technology Center (PSTNT), National Nuclear Energy Agency of Indonesia (BATAN), Bandung, West Java 40116, Indonesia; c Division of Radiation Protection and Safety Control, Cyclotron and Radioisotope Center (CYRIC), Tohoku University, Sendai 980-0845, Japan; d Department of Biology Pharmacy, Faculty of Pharmacy, Padjadjaran University, Sumedang, West Java, 45363, Indonesia; e Biotechnology Master Program, Faculty of Postgraduate, Padjadjaran University, Bandung 40132, Indonesia

**Keywords:** Breast cancer, Estrogen receptors β, Genistein, Technetium-99 m, Physicochemical properties, Thermodynamic stability, Density functional theory

## Abstract

**Objective:**

Genistein is an isoflavone molecule with a high affinity for estrogen receptors (ER), which could lead to the mechanism of selective estrogen receptor modulators (SERMs) in breast cancer. Genistein labeling with technetium-99^m^ can be a new promising strategy for diagnostic breast cancer. In this research, we evaluate the physicochemical characteristics of the [^99m^Tc]Tc-genistein complex and describe the optimal labeling method parameters. We also calculated density functional theory to study the stability constants to support complex formation analysis (DFT).

**Methods:**

The genistein was directly labeled with ^99m^Tc, and its stability as well as its potential for usage as a radiotracer were all investigated. DFT calculations with thermodynamic cycles to determine chemical coordination models and calculate thermodynamic constants of complex more accurately.

**Results:**

The radiochemical purity of [^99m^Tc]Tc-genistein showed a high yield of 93.25% ± 0.30% and had good physicochemical properties. The stability of the Tc(IV)-genistein complex was confirmed by DFT calculations at a value of 99.0822.

**Conclusions:**

As a result, [^99m^Tc]Tc-genistein could be a potential radiotracer kit for SPECT imaging of breast cancer.

## 1 Introduction

Genistein as an isoflavone compound has an important role in the mechanism of Selective estrogen receptor modulators (SERMs) because it has a high affinity for ERβ in target tissues and resist stimulation of the breast, bone, and endometrium. Genistein has potential as a specific ligand for labeling with technetium-99^m^, making it a potential target or prognostic marker of breast cancer. The death rate caused by breast cancer globally in 2020 is 684,996 and this is predicted to increase every year [1]. Early detection of breast cancer, when it is small and has not spread, will be easier to treat successfully [2]. In addition, the sensitive method of detecting micrometastatic conditions allows therapy to be scaled up in a higher-risk subset of patients and avoids patients from potential unnecessary side effects of treatment [3].

Selection of alternative treatments and predictive factors for breast cancer prognosis that is currently widely used include estrogen receptor-positive (ER+), carcinoembryonic antigen (CEA), progesterone receptor (PR), human epidermal growth factor receptor 2 (HER2), urokinase plasminogen activator (uPA), plasminogen activator inhibitor 1 (PAI-1). The evaluation of clinical variables, such as nodal involvement, tumor size, histological type, tumor grade and surgical margins [4,5,6]. The active ER signal stimulates cell proliferation and accounts for 75% of all diagnosed breast cancers [7].

Genistein is an isoflavones compound that is abundantly found in soybean seeds with the chemical name [5,7-dihyroxy-3-(-4-hydroxyphenyl)-4H-1-benzopyran-4-one], shown to be potentially specific in the treatment of certain types of breast tumors [8]. Genistein shows a strong affinity for human estrogen receptor beta [9].

The development of new radiopharmaceutical imaging kits is important for the early diagnosis of breast cancer and patient survival. Currently, Technetium-99^m^ is widely used in nuclear medicine to perform single-photon emission computerized tomography (SPECT) in order to detect cancer due to its ideal characteristics, including its 6.02 h physical half-life, low cost, and low -radiation energy (0.1405 meV), as well as its ready availability [10,11,12,13].

**Fig. 1:**
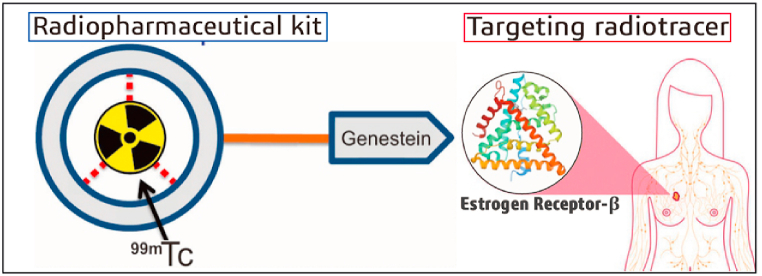
The general scheme of [^99m^Tc]Tc-genistein as a radiotracer for breast cancer.

This study provides information on [^99m^Tc]Tc-genistein which can be developed into a radiopharmaceutical kit for the detection of breast cancer patients specifically and accurately (Fig. 1). In accordance with the requirements for a potentially novel radiopharmaceutical, we developed an easier direct labeling method, requiring simple components with qualified radiochemical purity yields of [^99m^Tc]Tc-genistein, and evaluation of physicochemical properties.

The stability of metal complexes and ligands in radiopharmaceutical compounds plays an important role in regulating kinetic stability for their potential applications as drugs, diagnostic agents, and targeted radiotherapy. Rational design by considering the affinity and selectivity of metal ions to ligands is the main focus in determining the stability constant (*K*_*1*_) for the complexes at equilibrium conditions. In this work, we attempted to calculate the thermodynamic and kinetic stability of the Tc(lV)-genistein complex using basis sets, and an implicit solvation model. The addition of the solvation models as an approach to the typical conditions of radiosynthesis and the application of radiopharmaceutical compounds based on metal-ligand complexes *in vivo* [14].

## 2 Materials and methods

### 2.1 Materials

Genistein and SnCl_2_·2H_2_O was purchased from Sigma-Aldrich, and the solvents used were purchased from Merck. Instant thin layer chromatography-silica gel (ITLC-SG) (Agilent, Technologies), dose calibrator (Victoreen), Single Channel Analyzer (ORTEC). The technetium-99^m^ radioisotope was obtained from the Enviro Technetium-99^m^ Generator, manufactured by Enviro Korea Co., Ltd.

### 2.2 Methods

#### 2.2.1 Radiolabeling of [^99m^Tc]Tc-genistein

The formulation of all parameters, including genistein as the ligand, SnCl_2_·H_2_O as the reducing agent, pH level, and incubation time, were optimized in order to produce [^99m^Tc]Tc-genistein complex. In addition, sodium pertechnetate solution [^99m^Tc] with an activity 7.4–17 MBq was added in the process of forming [^99m^Tc]Tc-genistein. The TLC method is used to evaluate the radiochemical purity (RCP) of the complex formation, which must meet the USP requirement of >90% [15,16].

The radiochemical purity of [^99m^Tc]Tc-genistein is evaluated using the TLC method, which identifies ^99m^TcO_2_ and ^99m^TcO_4_^-^ impurities. This quality control method is simple and practical to use. The stationary phase used is TLC SGF-254 with a mobile phase of NaCl solution. This TLC system aims to separate TcO^4−^ impurities, where TcO^4−^ will migrate and [^99m^Tc]Tc-genistein compound will remain at the starting point [17].

^99m^TcO_2_ was separated using the stationary phase, ITLC-SG, and the mobile phase, a solution of C_2_H_5_OH, H_2_O, and NH_4_OH (2:5:1). As a result, the impurity of hydrolyzed ^99m^Tc (^99m^TcO_2_) will remain at the starting point, and the [^99m^Tc]Tc-genistein compound will migrate carried away by the mobile phase [17]. The strips were cut into small segments (0.5 cm) and measured for their radioactive activity using SCA (Single Channel Analyzer), and the calculation of RCP of [^99m^Tc]Tc-genistein is based on the equation:

(1) % [^99m^Tc]Tc-genistein = 100% - (% ^99m^TcO2 + % ^99m^TcO4-)

#### 2.2.2 Physicochemical properties study of the radioconjugates

##### 2.2.2.1 Radioconjugates lipophilicity test

Lipofolicity of labeled compounds is defined as the result of the calculation of the decimal logarithm (log P) of the partition coefficient (P) between two immiscible phases, measured in PBS solution (aqueous phase, polar phase, pH 7.4), and n-octanol solvent (organic phase, solvent non-polar) with the equation [18].

(2) $$\log\;P=\log\frac{Ao}{Aw}$$

where Ao—organic phase radioactivity; Aw—aqueous phase radioactivity.

The test was conducted by adding 2 mL of 1-octanol solution and 2 mL of 0.9% NaCl (pH 7.4) into centrifuge tubes and adding 10–50 μL of [^99m^Tc]Tc-genistein solution. The solution was homogenized by vortexing for 1 min and centrifuged at 3000 rpm for 10 min. Each 100 μL of the n-octanol fraction and saline solution was counted for radioactivity by SCA. The partition coefficient was determined by calculating the radioactivity ratio of n-octanol and saline solution.

##### 2.2.2.2 Stability of ^99m^Tc-labeled genistein

The *in vitro* stability of the [^99m^Tc]Tc-genistein in an aqueous solution was determined at room temperature. Measurements will be observed the percentage of radiochemical purity by TLC every 1 h for 5 h of observation time and its physical appearance [16].

##### 2.2.2.3 Measurement of plasma protein binding

Determination of plasma protein binding was carried out by precipitation method using TCA solution. A total of 500 μL of blood samples were added to 50 μL of radiopharmaceutical [^99m^Tc]Tc-genistein and homogenized by vortex for 1 min. The mixture was incubated for 10 min at 37 °C, then 1 mL of 0.9% NaCl solution and 1 mL of 5% TCA solution were added. This solution was centrifuged at 3000 rpm for 15 min. The precipitate formed is then separated from the supernatant. The supernatant solution was added again with 1 mL of TCA solution, the process of precipitation and separation was repeated. The precipitate fraction was washed again with the addition of 1 mL of 0.9% NaCl solution, centrifuged and separated again [19]. Radioactivity of the precipitate fraction (a) and the supernatant (b) will be measured by SCA and the plasma protein binding value is calculated by the following equation.

This study has been reviewed and approved by the Ethics Committee of the National Nuclear Energy Agency Indonesia with the number: 002/KEPHP-BATAN/IV/2021.

(3) $$plasma\;protein\;binding\;\left(\%\right)=\frac{a}{\left(a+b\right)}\;x\;100\%$$

#### 2.2.3 Predicting stability complex Tc(IV)-genistein using DFT method

This computational chemistry method was started by calculating the chemical parameters of the genistein ligand structure, including the analysis of NPA (natural population analysis), Natural Bond Orbital Analysis (NBO), and frontier molecular orbitals by a Small Highest Occupied Molecular Orbital-Lowest Unoccupied Molecular Orbital (HOMO-LUMO). Ligands will coordinate with the core (Tc^4+^ core) providing the most stable structure with six-coordinate. This structure is designed with the oxidation stability of technetium in mind and provides a stable pharmacokinetic profile of the geometric complex [20]. Structural optimization was carried out on [Tc(H_2_O)_6_]^4+^, genistein ligand, and complex [Tc(IV)(genistein)(H_2_O)_4_]^3+^ complex. Optimization and frequency of each structure were done using Gaussian 16.0 software.

In this study, we focus on predicting the thermodynamic stability of the formation Tc(lV) complexes with genistein ligands for a ratio of 1: 1. The calculation of metal/ligand complexes 1:1 at equilibrium conditions M + L **⇌** ML, where the value of the stability constant K_1_ for the reaction in solution correlates with the change in free energy - Gibbs, ΔG_aq_ with the equation:

(4) $$\mathrm{Log}\;K1=\frac{-\Delta G_{aq}}{2.303\;RT}$$

**Fig. 2:**
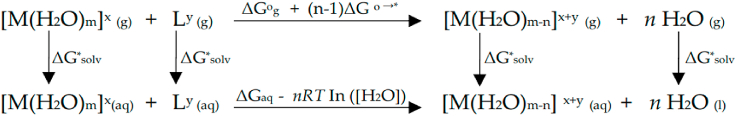
Thermodynamic cycle used to determine ΔG_aq_.

The principle for determining ΔG_aq_ is based on the thermodynamic cycle shown in Fig. 2.

In this process, ΔG°_g_ is the change in free energy required by the metal and ligand bonds in the gas phase, and ΔG*_solv_ to describe the free energy of solvation for the change of 1 mol of solute from the gas phase to the aqueous phase [21]. Note that ΔG°_g_ is determined under the standard ideal gas at 1 atm (24.46 mol/L) to 1 M (1 mol/L) by the equation:

ΔGo →* = -T ΔS 0 →* = RT In (Vo/V*) = R.T.In (24.46) = 1.89 kcal/mol (T = 298.15)

Correction calculations are required G_aq_* = G_aq_* + RT ln ([H_2_O]), to represent the state of the system when pure solvent H_2_O_(l)_ is used as the reference state for the solvent. The value of *R.T*.ln([H_2_O]) = 2.38 kcal/mol describes the free energy change associated with the displacement of the solvent from the standard solution phase concentration of 1 M to the standard pure liquid state, 55.34 M [22]. Using the gas-phase geometries, we calculated single-point aqueous solvation free energies, ΔG*solv, with the application of the solvation model (SMD). We used M06/6-311 + G (d) as the functional/basis sets.

## 3 Results

### 3.1 Radiolabeling of [^99m^Tc]Tc-genistein

The physical appearance of the labeled compound was noted transparent colorless liquid with no odor. The radiochemical purity of [^99m^Tc]Tc-genistein, determined by a simple TLC method gave an RCP yield of 93.25% ± 0.30%. Optimum labeling conditions obtained concentration of SnCl_2_·2H_2_O solution was 15 mg/mL, genistein concentration 10 mg/mL, optimum pH at 8, and incubation time of 10 min (Supplementary Tables S1-S3).

### 3.2 Physicochemical properties study of the radioconjugates

#### 3.2.1 Stability evaluation at room temperature

The stability of ^99m^Tc-genistein was evaluated by monitoring radiochemical purity (RCP) at different time points (10, 60, 120, 180, 240, 300 min) by TLC method. The RCP value of the preparation as well as physicochemical changes will be determined by this test. The assay is run for 5 h following the incubation period, and it is based on the ^99m^Tc half-life of 6.01 h. The stability results showed that the [^99m^Tc]Tc-genistein preparation can be developed as a radiopharmaceutical kit with an RCP >90% for 2 h and a clear physical appearance until the fifth hour (Table 1).

**Table 1:** The stability test of [^99m^Tc]Tc-genistein at room temperature.

Time (min)	Impurities (%)		RCP (%) [^99m^Tc]Tc-genistein
	^99m^TcO_2_	^99m^TcO_4_	
**10** **60** **120** **180** **240** **300**	1.33 ± 0.3 1.70 ± 0.5 2.55 ± 0.42 4.51 ± 1.96 8.76 ± 2.37 7.05 ± 2.94	5.66 ± 0.4 6.28 ± 0.12 6.90 ± 0.16 8.67 ± 1.84 10.48 ± 2.58 17.98 ± 2.08	93.01 92.02 90.55 86.82 80.76 74.97

#### 3.2.2 Lipophilicity value

Predicts drug permeability in cell/organ membranes and the polarity of the molecule being tested to evaluate its pharmacokinetic and pharmacodynamic characteristics. Drug molecules with high lipophilicity character have high affinity for fat and low affinity for water; they are also able to effectively cross cell membranes. Pharmacologically, lipophilicity plays an important role as a prognostic factor of a drug and other medical preparations to predict toxic effects and biological activities, their accumulation in organisms, and metabolism of substances. Lipophilicity according to Lipinski rules of 5 (Ro5) with a value of log P < 5 [23,24]. The results of the lipophilicity of the [^99m^Tc]Tc-genistein had a log P value of −0.77134 ± 0.12.

#### 3.2.3 Plasma protein binding

The precipitation method was used to measure how much [^99m^Tc]Tc-genistein was bound to plasma proteins. The protein precipitation agent in this procedure is a 5% TCA solution. The plasma protein binding score obtained was 75.29% ± 2.59%, indicating that this complex has good protein binding affinity.

### 3.3 Predicting stability complex Tc(IV)-genistein using DFT method

All calculations were conducted by implementing density functional theory (DFT) as applied to Gaussian 16, and the graphical visualization of structure using ChemCraft program. The optimization of the structure and frequency calculations were performed out in the gas phase. Geometry optimization of each structure and frequency calculations were carried out at M06/6-311 + G (d). The frequency calculation aims to verify that all geometric structures have reached the minimum energy conditions from the potential energy surface and to calculate the thermal correction of the enthalpy and total entropy at the ideal gas temperature [25].

**Fig. 3:**
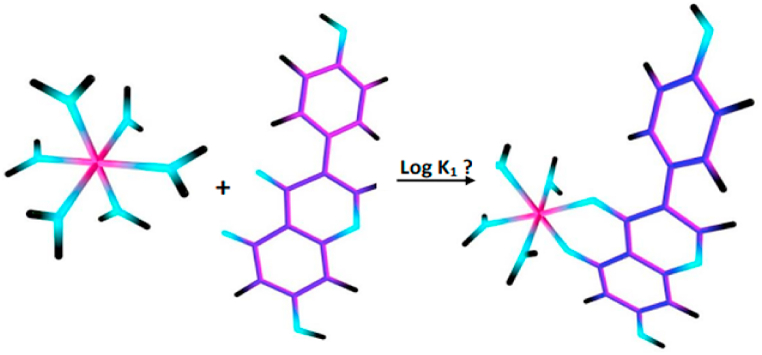
Representative equilibrium geometries of the Technetium ion with 6 coordinated water molecules, [Tc(H_2_O)_6_]^4+^, [Genistein]^1−^ ligand, and their complex [Tc(lV)(genistein)(H_2_O)_4_]^3+^.

The NPA and HOMO-LUMO analysis concluded that O atoms number 2 and 3 are the most potential regions to bond with metal atoms (Tc^4+^) (Figure S1). After obtaining the best structural model of the [Tc(genistein) (H_2_O)_4_]^3+^ complex, we performed geometry optimization and DFT calculations. The structural model of the complex [Tc(genistein) (H_2_O)_4_]^3+^ calculated by DFT, and complex cartesian coordinates under equilibrium geometry conditions is shown in Figure S2. The representative equilibrium geometries of the technetium ion with six coordinated water molecules, [Tc(H_2_O)_6_]^4+^, genistein-1 ligand, and their complex [Tc(genistein)(H_2_O)_6_]^4+^ (Fig. 3).

DFT computations utilizing computationally demanding techniques like implicit solvent quantum can greatly improve predictions of the absolute stability constants. Finally, in Table 2, we found that the selection of the basis sets (6-311 + G(d)), density functionals (M06), the models for solvation that were chosen (SMD) give the best stability constants (log K_1_) at DMSO solvent is 99.0822.

**Table 2:** Stability constants (log K_1_) for the Tc(IV) genistein complex.

Equilibrium	Functional/Basis Sets	log K_1_ Water	log K_1_ DMSO	log K_1_ Ethanol	log K_1_ Methanol
Tc^4+^ + (genistein)^1−^ ⇌ [Tc(IV) (genistein)]^3+^	M06/6-311 + G(d)	39.7215	99.0822	52.3161	44.676

## 4 Discussion

Estrogen receptors have become a major focus in treatment and predicting factors for breast cancer prognosis. Genistein with high binding affinity for ERβ targets will be able to respond to active ER signals in stimulating cell proliferation so that it can improve the sensitivity for detecting breast cancer and its distant metastases [9].

In this study, we investigated the optimum conditions for labeling genistein with technetium-99^m^ and evaluated the physicochemical properties of the [^99m^Tc]Tc-genistein complex. DFT calculations with solvation models can provide accurate information about the stability of the complex formed.

The labeling of genistein with ^99m^Tc was successful with the single-step direct labeling method providing high radiochemical purity. Moreover, the complex formed can be monitored relatively simply and easily with the TLC method. These results indicate that the radiopharmaceutical formulation has met the requirements of the United State Pharmacopeia, with a clear, colorless solution [15]. Furthermore, this RCP value is predicted to be able to reach the target organ well so that it can emit photons to be detected on a gamma camera.

[^99m^Tc]Tc-genistein turned out to be a highly with log P values of −0.77134 ± 0.12. These values indicate that this compound can pass through the absorption mechanism well, with less active penetration into cells. Therefore, this log P result still complies with Lipinski's rule [18].

The room temperature stability of the [^99m^Tc]Tc-genistein showed RCP values > 90% up to 2 h. However, after 2 h of storage at room temperature, the RCP value of the compound decreased due to the increase in impurity values of TcO_4_^−^ and TcO_2_. This could be due to the reduced ^99m^Tc, which has not been bound to genistein, can return to its original form because the reaction is irreversible. The stability results also showed that the [^99m^Tc]Tc-genistein had the appearance of a clear, colorless, and particle-free solution for up to 5 h of observation.

Plasma protein binding provides important information regarding the character of unbound drugs *in vivo*. The [^99m^Tc]Tc-genistein complex showed high plasma protein binding of 75.29% ± 2.59%. This result is relatively the same as the study conducted by [^14^C]C-Genistein 77.3 ± 6 4.7% [26]. This condition can confirm that the genistein complex has good protein binding affinity and explains that genistein remains *in vivo* for a relatively long time because the bound site can serve as a reservoir or depot for slowly releasing genistein as an unbound form [27].

The DFT calculation of the [Tc(IV) (genistein)]^3+^ complex was carried out with a thermodynamic cycle designed to largely cancel the systematic error in the calculation of the free energy change in the liquid phase, ΔG_aq_, and the stability constant, log K_1_. The first DFT calculation step is to find the atomic position on the ligand that will bind to the metal to form the most stable configuration of the complex structure. NPA and NBO analysis of the genistein ligand structure is useful for understanding electron density delocalization, and for measuring intermolecular or intramolecular interactions. This information can ensure the charge transfer in the chelator complex is important because it affects the interaction of the radiometal ion with the ligand [21]. Furthermore, we also performed HOMO-LUMO analysis to provide the information the prediction of the most reactive positions and support the information on which reaction occur in the conjugated system [28]. The results showed that the O–H atomic bond between O-2 and H-28 atoms has the smallest bond order of 0.6497, compared to the bond between O-4 and H-30 (0.7515), and O-5 and H-29 bonds (0.7555), therefore the atomic bond of O-2 and H-28 is the deprotonation location which is the best position for interaction with [Tc(H_2_O)_6_]^4+^.

Incorporating chemically significant solute-solvent explicitly into the quantum chemical model is one method to increase the accuracy of solvation estimates for ions. In this study, we used the SMD model (solvation model density) which is a continuum solvation model that can be applied to any charged or uncharged solute in the solvent [21]. M06 of density functionals which has advantages in calculating main group thermochemistry, thermochemical kinetics, noncovalent interactions, excited states, and transition elements [29]. The solute-solvent interaction has a very strong and profound effect on chemical reactivity. Polar protic solvents and dipolar aprotic solvents have an important role in ionic chemical reactions because of their properties that dissolve ionic species such as methanol, dimethyl sulfoxide (DMSO), dimethylformamide (DMF) and acetonitrile. Genistein has good solubility in ethanol, methanol, and DMSO, but poorly soluble in water [30]. DFT calculation of [Tc(IV)(genistein)]^3+^ complex in various solvents showed significant results that the formation of the most stable complex in DMSO solvent, indicated by the highest value of stability constant (log K_1_) (99,0873) compared to other solvents.

## 5 Conclusions

The direct labeling method was applied to develop [^99m^Tc]Tc-genistein, and it was successfully used to achieve high radiochemical purity that fulfills the criteria. [^99m^Tc]Tc-genistein has good physicochemical characteristics which can potentially be developed into a radiotracer kit for breast cancer. We designed the representative equilibrium geometries of the technetium complex [Tc(IV)(genistein)]^3+^, and the DFT calculation obtained the most stable stability constant in the DMSO solvent. In the near future, we plan to do *in vivo* investigations to explore the profile of the [^99m^Tc]Tc-genistein complex.

## Author contribution statement

Danni Ramdhani: Conceived and designed the experiments; Performed the experiments; Contributed reagents, materials, analysis tools or data; Wrote the paper.

Nita Listiani, Regaputra S Janitra: Performed the experiments; Analyzed and interpreted the data.

Maula Eka Sriyani: Conceived and designed the experiments; Performed the experiments; Contributed reagents, materials, analysis tools or data.

Eva Maria Widyasari: Conceived and designed the experiments; Contributed reagents, materials, analysis tools or data.

Hiroshi Watabe, Resmi Mustarichie, Sri Agung Fitri Kusuma: Analyzed and interpreted the data; Wrote the paper.

## Funding statement

This research did not receive any specific grant from funding agencies in the public, commercial, or not-for-profit sectors.

## Data availability statement

Data included in article/supp. material/referenced in article.

## Declaration of competing interest

The authors declare no conflict of interest.

## References

[1] World Health Organization (WHO) (2020). https://who.int/data/gho/data/themes/mortality-andglobal-health-estimates/ghe-leadingcauses-of%20death.

[2] World Health Organization (2017).

[3] Heather P.A., Justin R., Sarah C.R., Gregory G., Priyanka R., Pedro E., Kan X., Christopher L., Tianyu L. (2020). Sensitive detection of minimal residual disease in patients treated for early-stage breast cancer. Clin. Cancer Res..

[4] Maggie U.C., Stephen K.C., David V., Dongxia G., Samuel L., Jacqueline S. (2009). Ki67 index, HER2 status, and prognosis of patients with luminal B breast cancer. J. Natl. Cancer Inst..

[5] Vallejos C., Gomez H., Cruz W.R., Pinto J.A., Dyer R., Velarde R.R., Suanzo J.F. (2010). Breast cancer classification according to immunohistochemistry markers: subtypes and association with clinicopathologic variables in a Peruvian Hospital Database. Clin. Breast Cancer.

[6] Petra M., Petar O., Sonja L., Slavko O., Katarina A., Lidija B.O. (2011). Tumor markers in breast cancereevaluation of their clinical usefulness. Coll. Antropol..

[7] Charles M.P., Therese S., Michael B.E., Matt V.R., Stefanie S.J., Christian A.R. (2000). Molecular portraits of human breast tumours. Nature.

[8] Martin J.R. (2016). Effects of soy containing diet and isoflavones on cytochrome P450 enzyme expression and activity. Drug Metab. Rev..

[9] Hua J., Jingjing F., Lin C., Pan H., Renbin L. (2018). The anticancer activity of genistein is increased in estrogen receptor beta l-positive breast cancer cells. OncoTargets Ther..

[10] Gann T., Chih H.C., Hsin E.W. (2009). Cancer nanotargeted radiopharmaceuticals for tumor imaging and therapy. Anticancer Res..

[11] Derya I.O., Makbule A., Hayal O., Ferda Y., Mine H.L., Semin A. (2016). Gamma scintigraphy and biodistribution of ^99m^Tc-cefotaxime sodium in preclinical models of bacterial infection and sterile inflammation. J. Label. Compd. Radiopharm..

[12] Geo F.B., Karen C.C., Janet S.B., Stephen A.M. (1998). Jawetz, Melnick and Adelberg’s Medical Microbiology; Appleton-Lange: Stanford.

[13] Rennen H.J., Boerman C., Oyen W.J., Corstens F.H. (2001). Imaging infection/inflammation in the new millennium. Eur. J. Nucl. Med..

[14] Ramdhani D., Listiani N., Sriyani M.E., Eva M.W., Mustarichie R., Watabe H. (2021).

[15] United States Pharmacopeial Convention (2005).

[16] Zolle I. (2007).

[17] Owunwanne A., Patel M., Sadek S. (2012).

[18] Michael J.W. (2010). Lipophilicity in drug discovery. Expet Opin. Drug Discov..

[19] Katarzyna M., Ewa W., Pawel K.H., Dagmara T., Aleksandra M., Synthesis G. Ewa (2022). Physicochemical and biological study of Gallium-68 and Lutetium-177-labeled VEGF-A165/NRP-1 complex inhibitors based on peptide A7R and branched peptidomimetic. Pharmaceutics.

[20] Mazzi U., Nicolini M., Bandoli G., Refosco F., Tisato F., Moresco A., Duatti A. (1990). Technetium and Rhenium in Chemistry and Nuclear Medicine 3. Cortina International: Verona, Italy..

[21] Edouard M., Romain P., Baptiste S., Jean N.J. (2017). Estimation of solvation quantities from experimental thermodynamic data: development of the comprehensive compsol databank for pure and mixed solutes. J. Phys. Chem. Ref. Data.

[22] H Robert D., B Libero J. (2004). Prediction of formation constants of metal−ammonia complexes in aqueous solution using density functional theory calculations. Chem. Commun..

[23] Jay T., David C., Ryan G., Alison S., Leslie A.K. (2016). Key factors influencing ADME properties of therapeutic proteins: a need for ADME characterization in drug discovery and development. mAbs.

[24] Matthew P.D., Thomas M.P. (2013). The ABCD of clinical pharmacokinetics. Therapeutic Advan. Drug Safety.

[25] Osman I.O. (2017). DFT Study of the structure, reactivity, natural bond orbital and hyperpolarizability of thiazole azo dyes. Int. J. Mol. Sci..

[26] Coldham N.G., J Sauer M. (2000). Pharmacokinetics of [^14^C]Genistein in the rat: gender-related differences, potential mechanisms of biological action, and implications for human health. Toxicol. Appl. Pharmacol..

[27] Jianguo M., Gerrit S., Jaap V., Schellens J.H.M. (1996). Comparison of ethanol plasma-protein precipitation with plasma ultrafiltration and trichloroacetic acid protein precipitation for the measurement of unbound platinum concentration. Cancer Chemother. Pharmacol..

[28] K Kyoung H., Young K.H., Jaehoon J. (2005). Basis set effects on relative energies and HOMO–LUMO energy gaps of fullerene C_36_. Theor. Chem. Acc..

[29] Yan Z., Donald G.T. (2008). The M06 suite of density functionals for main group thermochemistry, thermochemical kinetics, noncovalent interactions, excited states, and transition elements: two new functionals and systematic testing of four M06-class functionals and 12 other functionals. Theoret. Chemistry Accounts.

[30] Jian G.W., Juan G., Yi-Ping Z., Yue Y., Xiao-Yu Z. (2010). Solubility of genistein in water, methanol, ethanol, propan-2-ol, 1-butanol, and ethyl acetate from (280 to 333) K. J. Chem. Eng. Data.

