# Supplementary material for: Estrogen receptor targeting with genistein radiolabeled Technetium-99m as radiotracer of breast cancer: Its optimization, characterization, and predicting stability constants by DFT calculation
Source: Heliyon. 2023 Jan 21;9(2):e13169. doi: 10.1016/j.heliyon.2023.e13169 (PMC9898673; doi:10.1016/j.heliyon.2023.e13169)
Supplement: Multimedia component 1 [file mmc1.docx]

*Supplementary Information for:*

**Estrogen receptor targeting with genistein radiolabeled Technetium-99^m^**

**as radiotracer of breast cancer: its optimization, characterization, and predicting stability constants by DFT calculation**

**Table S1.** The optimum pH and radiochemical purity of [^99m^Tc]Tc-genistein

| pH | ^99m^TcO_2_ | ^99m^TcO_4_^-^ | [^99m^Tc]Tc-genistein | Description |
| --- | --- | --- | --- | --- |
| 3 | 13.23 ± 0.46 | 3.38 ± 1.74 | 83.40 ± 2.08 | cloudy |
| 4 | 13.88 ± 0.77 | 1.46 ± 0.07 | 84.66 ± 0.89 | cloudy |
| 5 | 10.77 ± 0.66 | 3.83 ± 1.18 | 85.40 ± 1.56 | cloudy |
| 6 | 10.45 ± 1.20 | 3.59 ± 0.78 | 85.96 ± 1.65 | cloudy |
| 7 | 9.28 ± 0.58 | 2.09 ± 0.79 | 88.64 ± 1.13 | cloudy |
| 7,5 | 7.19 ± 1.94 | 2.98 ± 1.73 | 89.83 ± 3.01 | cloudy |
| 8 | 3.05 ± 0.45 | 4.98 ± 1.16 | 91.97 ± 1.43 | clear, yellowish |
| 9 | 7.59 ± 0.96 | 11.47 ± 1.16 | 80.94 ± 1.74 | clear, yellowish |
| 10 | 9.72 ± 0.40 | 9.90 ± 1.15 | 80.38 ± 1.40 | clear, yellowish |

**Table S2.** The optimum of SnCl_2_.2H_2_O and radiochemical purity of [^99m^Tc]Tc-genistein

| SnCl_2_·2H_2_O | ^99m^TcO_2_ | ^99m^TcO_4_^-^ | [^99m^Tc]Tc-genistein | Description |
| --- | --- | --- | --- | --- |
| 10 μl | 7.02 ± 1.15 | 5.36 ± 1.37 | 87.62 ± 2.06 | clear, yellowish |
| 20 μl | 8.08 ± 1.71 | 5.05 ± 1.74 | 86.87 ± 2.82 | clear, yellowish |
| 30 μl | 5.06 ± 1.13 | 4.10 ± 0.94 | 90.84 ± 2.38 | clear, yellowish |
| 40 μl | 8.76 ± 2.11 | 3.25 ± 0.94 | 87.99 ± 2.67 | clear, yellowish |
| 50 μl | 15.51 ± 1.63 | 4.26 ± 0.69 | 80.22 ± 2.05 | clear, yellowish |

**Table S3.** The optimum of genistein solution and radiochemical purity of [^99m^Tc]Tc-genistein

| Genistein (mg/mL) | ^99m^TcO_2_ | ^99m^TcO_4_^-^ | [^99m^Tc]Tc-genistein | Description | |  |
| --- | --- | --- | --- | --- | --- | --- |
| 1 | 3.49 ± 0.39 | 27.00 ± 0.37 | 69.51 ± 0.62 | | clear, yellowish | |
| 2 | 6.28 ± 0.19 | 13.99 ± 0.05 | 79.73 ± 0.23 | clear, yellowish | |  |
| 3 | 4.39 ± 2.52 | 10.13 ± 2.03 | 85.48 ± 3.74 | clear, yellowish | |  |
| 4 | 7.35 ± 0.58 | 4.71 ± 0.91 | 87.94 ± 1.25 | clear, yellowish | |  |
| 5 | 4.07 ± 1.28 | 3.74 ± 0.97 | 92.19 ± 1.86 | clear, yellowish | |  |
| 6 | 7.39 ± 0.58 | 2.67 ± 0.25 | 89.94 ± 0.73 | cloudy | |  |
| 7 | 8.86 ± 0.16 | 2.36 ± 0.75 | 88.78 ± 0.88 | cloudy | |  |
| 7.5 | 0.73 ± 0.52 | 5.34 ± 0.72 | 93.92 ± 1.03 | cloudy | |  |
| 10 | 0.83 ± 0.70 | 5.15 ± 0.70 | 94.02 ± 1.15 | cloudy | |  |

**
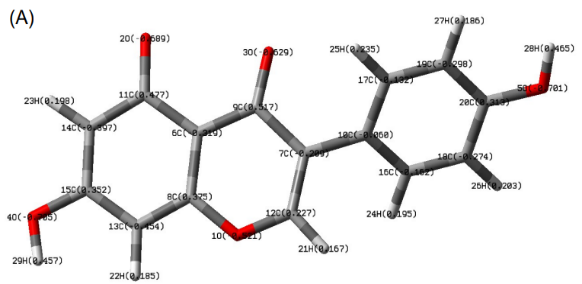

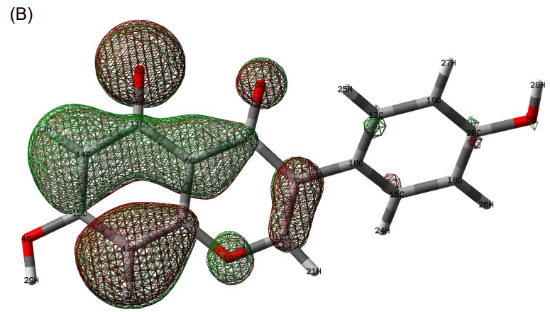
 Figure S1**. The structure of genistein ligands is based on: (a) NPA (natural population analysis); (b) Surface Contour Analysis HOMO (highest occupied molecular orbital)


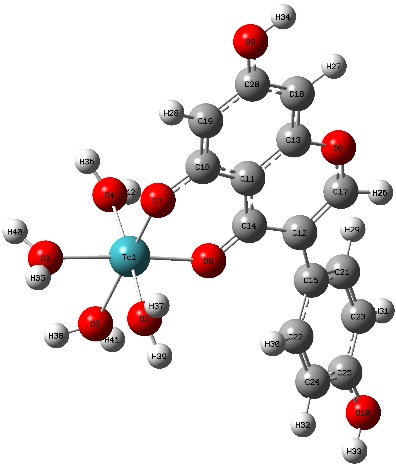


**Figure S2.** DFT-optimized structure and cartesian coordinate of [Tc(lV)(genistein)(H_2_O)_4_]^3+^ complex

**Cartesian Coordinates (Angstroms)**

Atom X Y Z

Tc 1.32076500 -1.85164900 0.10423400

O 1.32402100 -2.38935500 -1.98246300

O 2.91645000 -3.30583400 0.17498500

O 1.42141600 -1.39328100 2.17525800

O 0.07602400 -3.60306900 0.51183300

O -0.35609500 3.49516800 0.16108200

O 2.45727100 -0.37661300 -0.34805800

O -0.28118400 -0.57570800 0.00680800

O 4.31175100 3.84687800 -0.32245400

O -6.71553600 -0.47581200 -0.28197600

C 0.92767700 1.47470100 -0.06076400

C -1.52330000 1.42773700 0.09870800

C 0.85572600 2.88977100 0.00936000

C -0.26080700 0.70686700 -0.00182400

C -2.84440400 0.82681700 0.06335600

C 2.24704600 0.93247600 -0.22830000

C -1.45715900 2.79978100 0.19401900

C 1.93452200 3.72730700 -0.06275600

C 3.34261000 1.75867800 -0.31399000

C 3.20407800 3.15249100 -0.23252900

C -3.90965500 1.40572400 0.81069600

C -3.14146200 -0.27531300 -0.78905100

C -5.18515300 0.94418600 0.70084400

C -4.41845300 -0.73096200 -0.93690800

C -5.46947700 -0.12163200 -0.19792300

H -2.33420300 3.43897500 0.26167300

H 1.78810500 4.80272400 0.00419200

H 4.33334400 1.33551600 -0.45340100

H -3.70632600 2.20697700 1.51672700

H -2.34625600 -0.71582000 -1.38107400

H -6.00702200 1.36319300 1.27586500

H -4.64871800 -1.53312800 -1.63553500

H -6.91122400 -1.19043900 -0.91198700

H 4.19716000 4.80765200 -0.26695500

H 3.29487900 -3.66255700 -0.64150600

H 2.23302300 -1.27623100 2.68653800

H 1.89606400 -1.89461900 -2.58679900

H 0.45299200 -4.47921400 0.67414300

H 0.55549500 -2.70778200 -2.47360100

H 3.54772400 -3.45874900 0.89079300

H -0.85203400 -3.61134300 0.77686400

**Table S4.** Energy terms from DFT calculations

| Complex | Functional/  Basis Sets | Total DFT  Energy (a.u.) | Thermal Correction to  Enthalpy (kcal/mol) | Total Entropy  (cal/mol/K) | G°g  (kcal/mol) |
| --- | --- | --- | --- | --- | --- |
| H2O | M06/ | -76,4035 | 0.025293 | 45.058 | -76,3997 |
|  | 6-311+G(d) |  |  |  |  |
| (Genistein)^1-^ | M06/ | -952,8098 | 0.222850 | 130.222 | -952,649 |
|  | 6-311+G(d) |  |  |  |  |
| [Tc(H_2_O)_6_]^4+^ | M06/ | -537,3715 | 0.168913 | 108.988 | -537,254 |
|  | 6-311+G(d) |  |  |  |  |
| [Tc(genistein)(H_2_O)_4_]^3+^ | M06/ | -1338,2682 | 0.342336 | 182.149 | -1338,01 |
|  | 6-311+G(d) |  |  |  |  |

^a^ 6-311+G(d) basis set was used for all atoms except Tc.

**Table S5.** DFT calculations of ΔG_solv_  (SMD)

| Complex | Water (kcal/mol) | DMSO (kcal/mol) | Metanol (kcal/mol) | Etanol (kcal/mol) |
| --- | --- | --- | --- | --- |
| H_2_O | -10,643 | -5,965 | -10,631 | -10,113 |
| (Genistein)^1-^ | -71,273 | -60,248 | -72,113 | -70,303 |
| [Tc(H_2_O)_6_]^4+^ | -833,383 | -695,735 | -825,006 | -794,665 |
| [Tc(genistein)(H_2_O)_4_]^3+^ | -369,328 | -311,003 | -368,975 | -347,885 |

**Table S6.** Compiled ΔG°g, ΔGaq, calculated in this work

| Equilibrium | Functional/  Basis Sets^a^ | ΔG°g  (kcal/mol) | ΔGaq (Water)  (kcal/mol) | ΔGaq (DMSO)  (kcal/mol) | ΔGaq (Methanol)  (kcal/mol) | ΔGaq (Ethanol)  (kcal/mol) |
| --- | --- | --- | --- | --- | --- | --- |
|  |  |  |  |  |  |  |
| [Tc(genistein)(H_2_O)_4_]^3+^ | M06/ | -570,1305 | -54,1954 | -135,1861 | -61,3531 | -71,3792 |
|  | 6-311+G(d) |  |  |  |  |  |

^a^ 6-311+G(d) basis set was used for all atoms except Tc.
